# What do otolaryngologists want to learn? An educational targeted needs assessment study^☆^

**DOI:** 10.1016/j.bjorl.2018.12.001

**Published:** 2018-12-31

**Authors:** Mustafa Daloğlu, Mustafa Kemal Alimoğlu

**Affiliations:** Akdeniz University, Faculty of Medicine, Department of Medical Education, Antalya, Turkey

**Keywords:** Otolaryngology, Needs assessment, Education, Residency, Otorrinolaringologia, Avaliação das necessidades, Educação, Residência

## Abstract

**Introduction:**

Targeted needs assessment which includes identifying the needs of learners is a key step of program development. However, this step is commonly underestimated in postgraduate medical education programs, including otolaryngology residency training. Determining the needs of otolaryngologists may help educators to design more purposeful continuing medical education training programs. Furthermore, needs of specialists may provide a clearer insight about effectiveness of the residency programs in that specialty.

**Objective:**

To determine training needs of otolaryngology specialists and to identify deficiencies in otolaryngology residency training programs.

**Methods:**

Seventy-eight otolaryngology specialists, who completed all data gathering forms properly, were included in this descriptive, cross-sectional study. Demographic data of the participants were collected. Training needs of the participants were determined in seven basic areas of otolaryngology via two-round Delphi method. The basic areas were otology–neurotology, rhinology, laryngology, head and neck surgery, pediatric otolaryngology, sleep disorders and facial plastic surgery. Additionally, we asked an open-ended question to investigate the reasons why the participants perceived themselves incompetent and undereducated, or why they needed further training in some of the basic otolaryngology areas.

**Results:**

Facial plastic surgery, otology-neurotology and head and neck surgery were the most cited training areas in the needs assessment. Training needs differed according to experience and place of work. Financial expectations, deficiencies in residency training, regression in knowledge and skills, and special interest were effective determinants on decisions of the participants while determining their training needs.

**Conclusion:**

Otolaryngologists need further training in some areas of their field due to different reasons. Determining these areas and reasons will help in designing more effective continuous medical education activities and residency training programs in otolaryngology.

## Resumo

**Introdução:**

A avaliação de necessidades específicas, que inclui a identificação das necessidades dos alunos, é um passo fundamental no desenvolvimento de programas educacionais. No entanto, essa etapa costuma ser subestimada em programas de pós-graduação em educação médica, inclusive na residência em otorrinolaringologia. Determinar as necessidades dos otorrinolaringologistas pode ajudar os educadores a projetar programas mais direcionados de treinamento em educação médica continuada. Além disso, a análise das necessidades dos especialistas pode oferecer uma visão clara sobre a eficácia dos programas de residência nessa especialidade.

**Objetivo:**

Determinar as necessidades de treinamento de especialistas em otorrinolaringologia e identificar deficiências nos programas de residência em otorrinolaringologia.

**Método:**

Este estudo descritivo e transversal incluiu 78 especialistas em otorrinolaringologia que preencheram todos os formulários de coleta de dados adequadamente. Os dados demográficos dos participantes foram coletados. As necessidades de treinamento dos participantes foram determinadas em sete áreas básicas da otorrinolaringologia com o método Delphi em duas rodadas. As áreas básicas foram otologia/neurotologia, rinologia, laringologia, cirurgia de cabeça e pescoço, otorrinolaringologia pediátrica, distúrbios do sono e cirurgia plástica facial. Além disso, uma pergunta aberta foi usada para investigar os motivos pelos quais os participantes consideravam ter pouco conhecimento ou ser incapazes de atuar nessa área, ou por que precisavam de mais treinamento em algumas dessas áreas básicas.

**Resultados:**

Na avaliação das necessidades, cirurgia plástica facial, otologia-neurotologia e cirurgia de cabeça e pescoço foram as áreas de treinamento mais relatadas. As necessidades de treinamento variaram de acordo com a experiência e o local de trabalho. Expectativas financeiras, deficiências no treinamento durante a residência, regressão do conhecimento e habilidades, além de interesse especial, foram determinantes efetivos nas decisões dos participantes ao identificar suas necessidades de treinamento.

**Conclusão:**

Por diferentes motivos, os otorrinolaringologistas precisam de mais treinamento em algumas áreas. A determinação dessas áreas e motivos ajudará a planejar atividades de educação médica continuada e programas de treinamento em residência em otorrinolaringologia mais eficazes.

## Introduction

According to a survey conducted in the UK, 17% of referrals in primary care in the adult population and 50% in the pediatric population are made to otolaryngologists.^1^ This shows the importance of undergraduate and postgraduate education in the field of otolaryngology in terms of community health.

In early 2000s, the Turkish Association of Otorhinolaryngology-Head and Neck Surgery (TAO-HNS) started studies to develop a core curriculum for 5 year otolaryngology residency training in Turkey. Goals and objectives of the sample core residency training curriculum were set considering the healthcare needs of the community. The association has also provided suggestions about educational strategies and implementation of the core residency curriculum.

In developing a medical education curriculum at any level, the six step approach suggested by Kern is a commonly referenced model. The steps are: (1) General needs assessment, (2) Targeted needs assessment, (3) Goals and objectives, (4) Educational strategies, (5) Implementation, and (6) Evaluation and feedback.^2^ When we tried to match the steps of Kern's model and history of developing a core otolaryngology residency training curriculum in Turkey, we found some attempts that could be placed in every step except for one. The ultimate goal of any undergraduate or postgraduate medical education curriculum is to solve health problems of the community. Therefore, the first step is general needs assessment which includes identifying the health care problems that will be addressed by the curriculum. While setting goals and objectives (the third step in the model) the TAO-HNS took commonly seen Ear, Nose and Throat (ENT) diseases in the Turkish population into account as an attempt for the first step (general needs assessment). TAO-HNS also suggested some educational strategies (Step 4), implementation (Step 5) and evaluation (Step 6) methods for the departments. For example, in order to achieve minimum acceptable standards nationwide, resident log-books were prepared by the association to be used in every department.^3^ However, the second step, “targeted needs assessment”, which includes identifying the needs of learners, has been ignored, i.e. learning needs of the otolaryngology residents have not been studied to date. Self-directed learning is a commonly preferred method for medical residents to learn throughout residency training process. Therefore, determining learning needs, special areas of interest or personal expectations may help educators support the professional development of the resident.^4^

The TAO-HNS also offers Continuing Medical Education (CME) activities for otolaryngology specialists. Targeted needs assessment lacks in the development process of such programs as well. Adult learning theory assumes that adults are motivated to learn as they experience needs and interests that learning will satisfy; therefore, these are the appropriate starting points for organizing adult learning activities.^5^ Consequently, it is clear that needs assessment of the target learner group, in other words, the process of identifying the gap between the current and ideal situation, is an important step in curriculum development and deserves every kind of effort. Although the concept and value of needs assessment is well accepted, relevant literature-based information in postgraduate medical education is limited.^6^

This study focuses on needs assessment of the specialists with the research question “What are the learning gaps and training needs of otolaryngology specialists?” Our expectation was that the answers to this question may also provide some indirect information about the learning needs of the residents who are the specialists of the near future. Therefore, the aim of this study was to determine training needs of otolaryngology specialists and to identify areas for further emphasis in the otolaryngology residency training programs.

## Methods

### Study design

This descriptive, cross-sectional study was performed among otolaryngology specialists to determine training needs of the participants for seven previously determined basic areas of otolaryngology diseases via two-round Delphi method. Additionally, we investigated the reasons behind why the participants perceived themselves incompetent and undereducated or why they needed training in some of the basic areas of otolaryngology.

### Participants and ethical issues

Our target population was the entire otolaryngology specialty in Turkey. However, considering the difficulties in reaching all of them, we preferred to study a sample population that may represent different ages, experience, careers or affiliation groups. We took the national congress as an opportunity to reach and encounter as much specialists as possible. We delivered the data gathering forms to 118 persons. Finally, 78 specialists who completed all data gathering forms properly in all rounds composed the study group. Ethical approval for the study was granted by Akdeniz University Board of Ethics on Noninvasive Clinical Human Studies (Reference No. 06.10.2016/515).

### Data gathering forms and process

We developed a written data form including three parts: (1) Demographic data (asking for age, gender, duration of experience as a specialist, and the institution the participant worked for); (2) A list to be ranked by priority order, and (3) Open ended questions. The list includes the titles of seven basic areas of otolaryngology: (1) Otology-Neurotology, (2) Rhinology, (3) Laryngology, (4) Head and Neck Surgery, (5) Pediatric Otolaryngology, (6) Sleep Disorders and (7) Facial Plastic Surgery. The list was generated regarding basic areas of continuing medical education activities called ENT schools which are organized by TAO-HNS.

Two-round Delphi technique was used to determine priorities of the participants in terms of training needs. The Delphi survey is a group facilitation technique, which is an iterative multistage process, designed to transform opinion into group consensus. It is a flexible approach that is used commonly within the health and social sciences.^7^ In our study we initially delivered the forms to the participants and asked them to complete the demographic part and order the list regarding the degree of training needs (or deficiencies). Giving a number between 1 (the area in which training is needed most) to 7 (the area in which training is needed least) to each item, the participants sorted the list content regarding their current competency levels, needs and frequently encountered problems. Retrieving the forms, we arranged the 7 item list regarding the priority levels given by the participants. We reversely scored the items marked by the participants, namely, we gave 7 points for the item which was scored 1 (top priority) by the participants. Then, we were able to arrange the list according o mean values of the items; the item with maximum mean value took part on the top of the new list. The new list that represented participant training priorities was redelivered (second round) to the participants 10 days later. We asked the participants to do the same task they did in the first round and reorder the list once again. After the second-round suggestions had been evaluated, we had a final list of training areas sorted by the participants regarding their priorities at two rounds.

The third part of the data gathering form was an open-ended question asking the reasons behind why the participants perceived themselves incompetent and undereducated or why they needed training in some of the basic areas of otolaryngology.

### Data analyses

We used descriptive analyses to calculate mean and median values of training need scores in each area. Since literature suggests that 10 years of practice is needed to reach ideal knowledge structure and clinical reasoning strategy for diagnostic accuracy and correct decision- making,^8^, ^9^ we divided the study group into 2 categories according to duration of the experience as a specialist in the field as 10 years or less and over 10 years. We used Student's *t* test to compare mean training need scores of these two experience groups in each of the seven areas.

We divided the study group into three categories according to the institutions they worked for as research and training hospitals (including university hospitals), state hospitals and private hospitals/practices. We used One Way ANOVA test to compare mean training need scores of these three groups in each of the seven areas. All statistical analyses were performed with IBM SPSS Statistics Version 20. The answers to the qualitative part of the data gathering form (3rd part) were categorized regarding main themes mentioned in the texts.

## Results

The mean age was 44.3 ± 12.1 (29–72) years and female/male ratio was approximately 1/2 in the study group. Mean duration of the experience as a specialist was 13.7 ± 11.2 (1–36) years. The distribution of the participants according to duration of the experience and work places can be seen in Table 1.

**Table 1:** Demographic characteristics of the study group.

Characteristics	*n*
*Age*	44.3 ± 12.1
*Gender*	
Female	27
Male	51

*Duration of experience as a specialist (years)*	
0–10	43
Over 10	35

*Institution*	
Training and Research Hospitals	24
State Hospital	35
Private hospital and practice	19

In the first round of Delphi implementation, seven training areas in the list were sorted regarding priorities of participants in the following order: (1) Facial plastic surgery, (2) Otology-neurotology, (3) Head and neck surgery, (4) Laryngology, (5) Sleep disorders, (6) Rhinology and (7) Pediatric otolaryngology. This order did not change with reevaluation of the ranking of the list by the participants in the second round (Table 2).

**Table 2:** The rankings mean and median scores of the areas in two Delphi rounds.

	1st round		2nd round	
	Mean ± SD	Median	Mean ± SD	Median
Facial plastic surgery	5.1 ± 2.3	6	5.1 ± 2.2	6
Otology-neurotology	4.6 ± 1.9	5	4.8 ± 1.9	5
Head and neck surgery	4.2 ± 1.9	4.5	4.4 ± 1.9	5
Laryngology	3.9 ± 1.5	4	3.8 ± 1.5	4
Sleep disorders	3.8 ± 2.0	4	3.8 ± 2.0	4
Rhinology	3.7 ± 1.9	4	3.6 ± 1.8	4
Pediatric ENT	2.7 ± 1.6	2	2.6 ± 1.6	2

In Table 3, mean training scores and ranks of each training area in experience groups of 10 years or less, and over 10 years were provided. The unique difference between mean training need scores of these two experience groups was found in sleep disorders area in favor of the group with experience over 10 years (Student *t* test, *p* = 0.003).

**Table 3:** Comparison of experience groups regarding training need scores in each area.

	≤10 years		>10 years		*p*^a^
	Rank	Mean ± SD	Rank	Mean ± SD	
Facial plastic surgery	1	5.4 ± 2.1	1	4.7 ± 2.4	0.143
Otology-neurotology	2	4.9 ± 1.8	2	4.6 ± 2.0	0.449
Head and neck surgery	3	4.5 ± 1.8	4	4.3 ± 1.9	0.778
Sleep disorders	6	3.2 ± 2.0	3	4.5 ± 1.6	0.003
Laryngology	4	4.0 ± 1.4	6	3.5 ± 1.7	0.220
Rhinology	5	3.6 ± 1.8	5	3.6 ± 2.0	0.965
Pediatric ENT	7	2.4 ± 1.3	7	2.8 ± 1.8	0.334

a Student t Test.

Table 4 provides mean training scores and ranks of each training area in participant groups working in different workplaces. Mean scores for the areas of facial plastic surgery, sleep disorders, laryngology and pediatric otolaryngology differ among the workplace groups (One Way ANOVA, *p* < 0.05 for all).

**Table 4:** Comparison of the training need scores of the groups working in different work places.

	Training/Research hospitals		State hospitals		Private hospitals and practices		*p*^a^
	Rank	Mean ± SD	Rank	Mean ± SD	Rank	Mean ± SD	
Facial plastic surgery	5	3.9 ± 2.5	1	5.8 ± 1.8	1	5.3 ± 2.2	0.006
Otology-neurotology	1	4.8 ± 1.8	2	4.9 ± 1.9	2	4.5 ± 2.0	0.708
Head and neck surgery	2	4.3 ± 2.3	3	4.7 ± 1.6	4	3.9 ± 1.9	0.279
Sleep disorders	3	4.2 ± 2.1	5	3.2 ± 1.8	3	4.4 ± 1.9	0.040
Laryngology	6	3.8 ± 1.2	4	4.2 ± 1.5	7	2.9 ± 1.6	0.007
Rhinology	4	4.1 ± 2.0	6	3.1 ± 1.5	5	3.8 ± 2.1	0.111
Pediatric ENT	7	2.8 ± 1.7	7	2.1 ± 1.3	6	3.2 ± 1.6	0.019

a One Way ANOVA.

Four themes emerged from the answers to the open-ended question about the reasons behind why the participants perceived themselves incompetent and undereducated, or why they needed training in some of the basic areas of otolaryngology. These themes were financial expectations (*n* = 31), deficiencies in residency training period (*n* = 44), regression in knowledge and skills throughout post-training period (*n* = 19), and special interest with the area (*n* = 40). Among the participants with financial expectations, most preferred (81%) training area was facial plastic surgery while it was head and neck surgery (47%) among those who explain the reason behind their training needs as knowledge and skill retention. The majority of the participants (47%) who needed training because of deficiencies in residency training period preferred facial plastic surgery, which is also most frequently preferred (38%) special interest area.

## Discussion

This study was conducted with the aim of determining training needs of otolaryngologists and identifying areas for further emphasis in the otolaryngology residency training programs. Facial plastic surgery, otology-neurotology and head and neck surgery were found to be the leading training areas that the participants perceived themselves incompetent and/or needed further training.

Health care professionals are expected to meet certain patient requirements as well as the use of the latest evidence-based approaches.^10^ As attention is focused on maintaining physician competency and eliminating medical errors, CME is becoming more highly regulated, and CME providers are being held to higher standards.^11^ When creating or revising a curriculum, targeted needs assessment is an integral step prior to developing appropriate goals and objectives.^2^ Teaching institutions and educators should be cognizant of the needs of learners.^12^ Targeted needs assessment for CME activities among specialists not only provides information about learner needs, but also helps evaluation of the residency programs from which those specialists graduated. In other words, common perception of incompetence and requirement of further training in some areas of the specialty may demonstrate the defects in residency training. As a commonly referred model, Kirkpatrick's Four Level Program Evaluation approach measures (1) The learner's reaction to the program (satisfaction), (2) The learning that takes place (confirmed by assessment process), (3) The change in behavior (transfer of the gained knowledge and skills to practice), and (4) Results achieved in service taking population.^13^ Our study results provide information for the third level of Kirkpatrick's program evaluation model by determining the areas of the residency training content that the participants felt themselves well or poor equipped for proper performance in real life.

Facial Plastic Surgery was at the top of the training needs list of our participants. Recently, there has been a growing interest in facial aesthetic procedures especially among young adults.^14^ Turkey is 9th in the world regarding aesthetic face and head procedures with 132,564 cases (3.1% of all cases worldwide) per year.^15^ If demand determines the supply, then it is clear that there is a serious amount of workload and pressure that otolaryngologists have to face in facial plastic surgery. This workload and/or pressure might be the reason for facial plastic surgery to be the most needed training area in our study. We know from the literature that higher income expectations play a motivating role in selection of training area among specialists.^16^ The increasing demand for aesthetic procedures may also be perceived as an extensive market with better income opportunities among otolaryngologists. This may be another factor carrying the facial plastic surgery to the top of the training need list. As a supporting data, we found that facial plastic surgery was the most preferred area (81%) among participants, who reported financial expectations as the main reason for their training needs. Finally, deficiencies in residency period might be another reason for top priority of facial plastic surgery. We found facial plastic surgery as the most preferred area among our participants, who reported deficiencies in residency period as the main factor in determining their training needs. Interest to facial plastic surgery was lowest among participants working in research and training hospitals, possibly due to the fact that such institutions, as tertiary care service providers, are more focused on challenging cases of the fields such as otology-neurotology or head and neck surgery.

Otology-neurotology was the second most preferred training area in our study group. This may be explained by significant technological developments in diagnosis and treatment of the diseases in the field.^17^ Besides the fast-growing theoretical knowledge, the number of procedural competencies is also multiplying. The complex nature of otology-neurotology procedures may lead to a prolonged learning processes. For this reason, the desired expertise level may be challenging in 5 year residency training program. Thus, inadequate residency training in the field of otology-neurotology might be the underlying reason for common training need in this area among our participants.

Physicians in all specialties care for gradually increasing number of cancer patients and survivors.^18^ This basically emphasizes the growing importance of head and neck surgery (third most needed training area in our study) among the other otolaryngology subspecialties. The programs of head and neck surgery rotations in residency training are usually structured intensely and the procedures are complicated and time- consuming. There are studies reporting that residents in head and neck surgery rotation have significantly fewer hours of sleep and higher hours of work per week compared to other rotations.^19^ Excessive workload of the residents may lead to burnout which could lower the quality of residency training via a motivational behavior, and it can also lower the quality of care for patients.^20^ So, our participants might have selected head and neck surgery as a prominent training need area since they were not well educated in the field during residency. Additionally, besides expertise, head and neck surgery also requires infrastructure and special equipment to be performed. The specialists working in hospitals that are not well-equipped for head and neck surgery naturally avoid the sophisticated and tiresome interventions of head and neck region in order to avoid any risk. This avoidance may lead to regression in both knowledge and surgical skills. We know that loss of competence occurs in even much simpler clinical skills in time if those skills are not performed after training.^21^ Consequently, loss of competence to some degree might be another reason for common need to head and neck surgery training. This point of view is also supported with the finding that head and neck surgery was the most preferred area (47%) among the participants who reported knowledge and skill regression as the main reason for their training needs.

Our participants with experience over 10 years reported more training needs in the area of sleep disorders compared to their less experienced colleagues. The reason might be associated with history of sleep medicine in Turkey. Sleep medicine has been the focus of interest in otolaryngology community especially in recent decade.^22^ Then experienced specialists in our study group were not trained in this field during their residency period, since sleep disorders were missing in their training curriculum. There should be some additional areas which are not missing but deficient in residency training periods of our participants. Thus, 56% of the study group pointed out deficiencies in residency training period as the main reason for training needs. Such deficiencies were reported in the literature. For example, Baugh et al. has shown that, despite the accreditation requirements on minimum numbers for key indicator procedures, residents have graduated without meeting these minimums.^23^

As seen in Table 4, interest level of the participants to some training areas differed according to the workplaces where they practiced. We have already mentioned the possible reason of less interest to facial plastic surgery among those working in training and research hospitals. However, less interest in sleep disorders and pediatric otolaryngology among participants who worked for state hospitals or low level of interest to laryngology among those working in private hospitals or practices are findings which may not be explained easily. These may be the areas, in which the participants may feel themselves highly competent, or patient profile may change depending on the institutions worked for and the physicians may not need any further training on rarely seen cases.

Further qualitative data would give valuable information about the reasons behind our findings. Therefore, shortage of qualitative part is the first limitation of the study. The second prominent limitation is about generalizability of the results. Results obtained from a limited number of otolaryngologists in a single country cannot be generalized.

## Conclusion

In conclusion, we found that facial plastic surgery, otology-neurotology and head and neck surgery were the leading training areas that the Turkish otolaryngologists perceived themselves inadequately prepared and/or needed further training. The underlying factors were problems with learning processes, financial and social preferences and personal experience and interest of the otolaryngologists. Better designed studies using a mix of qualitative and quantitative methods with larger populations from different countries and cultures are needed to have more reliable results. Insights from such studies will help planning more target-oriented CME activities for otolaryngologists and better structured training programs for residents.

## Conflicts of interest

The authors declare no conflicts of interest.

## Author's contributions

Both Daloglu M. and Alimoglu M.K. designed and performed the study; analyzed the data; wrote the manuscript. All authors read and approved the final manuscript.
